# Sensorimotor and inhibitory control in aging *FMR1* premutation carriers

**DOI:** 10.3389/fnhum.2023.1271158

**Published:** 2023-11-16

**Authors:** Heather Fielding-Gebhardt, Shannon E. Kelly, Kathryn E. Unruh, Lauren M. Schmitt, Stormi L. Pulver, Pravin Khemani, Matthew W. Mosconi

**Affiliations:** ^1^Life Span Institute, University of Kansas, Lawrence, KS, United States; ^2^Scholars Strategy Network, Boston, MA, United States; ^3^Kansas Center for Autism Research and Training (K-CART), University of Kansas, Lawrence, KS, United States; ^4^Division of Behavioral Medicine and Clinical Psychology, Cincinnati Children’s Hospital Medical Center, Cincinnati, OH, United States; ^5^Department of Pediatrics, University of Cincinnati College of Medicine, Cincinnati, OH, United States; ^6^Division of Autism and Related Disorders, Emory University School of Medicine, Atlanta, GA, United States; ^7^Movement Disorders Program, Swedish Neuroscience Institute, Seattle, WA, United States; ^8^Clinical Child Psychology Program, University of Kansas, Lawrence, KS, United States

**Keywords:** FXTAS, FMR1 premutation, antisaccade, inhibitory control, eye movements

## Abstract

Aging *FMR1* premutation carriers are at risk of developing neurodegenerative disorders, including fragile X-associated tremor/ataxia syndrome (FXTAS), and there is a need to identify biomarkers that can aid in identification and treatment of these disorders. While FXTAS is more common in males than females, females can develop the disease, and some evidence suggests that patterns of impairment may differ across sexes. Few studies include females with symptoms of FXTAS, and as a result, little information is available on key phenotypes for tracking disease risk and progression in female premutation carriers. Our aim was to examine quantitative motor and cognitive traits in aging premutation carriers. We administered oculomotor tests of visually guided/reactive saccades (motor) and antisaccades (cognitive control) in 22 premutation carriers (73% female) and 32 age- and sex-matched healthy controls. Neither reactive saccade latency nor accuracy differed between groups. *FMR1* premutation carriers showed increased antisaccade latencies relative to controls, both when considering males and females together and when analyzing females separately. Reduced saccade accuracy and increased antisaccade latency each were associated with more severe clinically rated neuromotor impairments. Findings indicate that together male and female premutation carriers show a reduced ability to rapidly exert volitional control over prepotent responses and that quantitative differences in oculomotor behavior, including control of visually guided and antisaccades, may track with FXTAS – related degeneration in male and female premutation carriers.

## 1 Introduction

Individuals who carry premutation repeat expansions (55–200 CGG repeats) on the first exon of the *FMR1* gene are at risk for age-related neurodegeneration that may result in motor and/or cognitive impairments. One such disorder is fragile X-associated tremor/ataxia syndrome (FXTAS), which is defined clinically by action tremor and/or gait ataxia (Hagerman et al., 2001, 2004). Radiological indicators include generalized brain atrophy, T2-weighted MRI hyperintensities of the middle cerebellar peduncles (MCP sign) (Jacquemont et al., 2003; Greco et al., 2006), and atrophy of the splenium of the corpus callosum (Brunberg et al., 2002; Apartis et al., 2012). Prevalence among male premutation carriers is relatively high, with 45% of male premutation carriers over 50 years experiencing FXTAS-related decline, and penetrance subsequently increasing with age (Jacquemont et al., 2004; Rodriguez-Revenga et al., 2009). In contrast, only 16% of female premutation carriers older than 50 years experience FXTAS, though there is a paucity of data regarding penetrance rates across older ages among females (Coffey et al., 2008; Rodriguez-Revenga et al., 2009). Multiple other co-occurring features of aging in *FMR1* premutation carriers that also may be present in both males and females include cognitive declines, peripheral neuropathy, psychiatric changes, and other health issues (Brega et al., 2008; Grigsby et al., 2008; Rodriguez-Revenga et al., 2010; Wong et al., 2014; Schmitt et al., 2022). Among the cognitive symptoms associated with aging are deficits in executive function which are particularly common (Brega et al., 2008; Cornish et al., 2009; Kraan et al., 2013a,b; Grigsby et al., 2014; Schneider et al., 2020; Moser et al., 2021; Storey et al., 2021). Declines in executive functions, including inhibitory control, in male and female premutation carriers are associated with FXTAS symptoms such that FXTAS patients demonstrate worse executive function than asymptomatic (FXTAS-) sex-matched premutation carriers and healthy controls (Grigsby et al., 2008; Yang et al., 2013; Grigsby et al., 2014; Yang et al., 2014). Also, executive dysfunction worsens with FXTAS severity (Schneider et al., 2020). Quantitative approaches for examining inhibitory control processes in aging premutation carriers are needed to detect subtle changes that may serve as early indicators of FXTAS or be useful for assessing target engagement and therapeutic response in clinical trials.

Oculomotor testing represents a promising quantitative approach for understanding phenotypes associated with the *FMR1* premutation. Oculomotor measures are sensitive to pathology in multiple diseases of aging (e.g., Parkinson’s Disease, Huntington’s Disease) suggesting that they may be valuable for detecting and tracking neurodegenerative processes (Gitchel et al., 2013; Shakespeare et al., 2015; Anagnostou et al., 2020; Waldthaler et al., 2022). Further, strong test–retest reliability of oculomotor testing has been demonstrated in both healthy aging populations (ICCs: 0.73–0.89) (Płomecka et al., 2020) and in individuals at risk for development of neurodegenerative disease (e.g., Huntington’s disease; ICCs: 0.83–0.87) (Blekher et al., 2009). Also, oculomotor indices are sensitive to change over time in both symptomatic individuals with neurodegenerative disease and pre-symptomatic individuals at risk for neurodegenerative disease relative to controls (Antoniades et al., 2010). Oculomotor paradigms can be used to examine multiple discrete neural systems including cortical-brainstem-cerebellar circuits supporting basic sensorimotor control such as movement precision and stable gaze fixation, and higher level cortical-striatal circuits involved in top-down executive processes, such as inhibition of prepotent responses (Pierrot-Deseilligny et al., 2003; Ploner et al., 2005; Hutton, 2008; Lennertz et al., 2012; Shelton et al., 2014). The neural substrates of oculomotor control are well understood based on human lesion, neuroimaging, and non-human primate studies, suggesting that clarifying oculomotor processes affected in aging premutation carriers may provide important new insights into neurodegenerative mechanisms of clinical decline (Krauzlis and Miles, 1998; Takagi et al., 1998; Desmurget et al., 2000; Pierrot-Deseilligny et al., 2002; Leigh and Kennard, 2004; Neggers et al., 2005; Leigh and Zee, 2006).

Multiple oculomotor behaviors may be affected in FXTAS based on studies showing both sensorimotor and executive impairments as well as pathology of key neural structures supporting basic oculomotor control (e.g., cerebellum, prefrontal cortex). Visual gaze fixation is an active process supported by cerebellar-brainstem circuits. Studying the stability of gaze fixation allows for identification of cerebellar and brainstem degeneration. Saccades are rapid eye movements made to shift gaze between points of fixation (Leigh and Zee, 2006). Square wave jerks (SWJ) are small intrusive horizontal saccades that disrupt visual fixation. They are present in healthy, aging, and pathological populations, but their frequency is increased in neurodegenerative motor diseases with brainstem pathology (Herishanu and Sharpe, 1981; Shallo-Hoffmann et al., 1989; Nowinski et al., 2005). Visually guided saccades are generated when novel stimuli are introduced into the observer’s environment, and their accuracy and consistency are modulated by cerebellar-brainstem circuitry and cortical-cerebellar feedback mechanisms. Reduced accuracy and increased accuracy variability across repeated movements may represent dysfunctions of cerebellar circuits involved in internal action representations guiding the precision of motor output and those involved in updating these representations based on error feedback information (Takagi et al., 1998; Robinson and Fuchs, 2001). The latency of exogenously driven saccades is supported by cerebellar-brainstem circuits involved in movement generation and movement cells in cortical (frontal and parietal eye fields) and subcortical (basal ganglia) circuits supporting action planning and initiation (Leigh and Zee, 2006). Prior studies of visually guided saccades have documented increased trial-to-trial accuracy variability in FXTAS+ (McLennan et al., 2022) and FXTAS- premutation males (Wong et al., 2014). In contrast, studies of female premutation carriers have provided mixed findings. One study demonstrated a significant increase in visually guided saccade latency between asymptomatic premutation females and controls (Moser et al., 2021), while another noted no differences between groups (Shelton et al., 2014).

Antisaccades are generated when an observer suppresses an exogenously driven reactive saccade in favor of generating a volitional saccade in the opposite direction of a suddenly appearing cue. Measurement of antisaccade latencies and error rates provide important indices of inhibitory control processes supported by fronto-striatal circuits. Antisaccade paradigms have demonstrated impairments in inhibitory control in premutation carriers. One study of antisaccade behavior demonstrated increased latencies in younger adult asymptomatic male premutation carriers relative to age-matched healthy controls (Wong et al., 2014), but another study did not report latency differences between asymptomatic male and female premutation carriers and controls in their 50s and 60s (McLennan et al., 2022). Additionally, male premutation carriers show both increased antisaccade latencies and elevated error rates associated with increased FXTAS symptom severity (McLennan et al., 2022). Asymptomatic adult female premutation carriers also appear to show increased rates of antisaccade errors relative to healthy female controls, though no known studies have examined antisaccade behavior in samples of female premutation carriers with symptoms of FXTAS (Shelton et al., 2014; Moser et al., 2021), thus limiting our understanding of how FXTAS symptoms may track with oculomotor performance in females.

The present study examined visually guided saccades and antisaccades in a primarily female sample of aging *FMR1* premutation carriers. Consistent with previous findings (Shelton et al., 2014; Wong et al., 2014; Moser et al., 2021; McLennan et al., 2022), we predicted that premutation carriers would demonstrate longer antisaccade latencies and higher rates of antisaccade errors relative to controls. We also predicted that premutation carriers would show greater inhibitory cost (i.e., lower efficiency in inhibiting unwanted saccades) (Wong et al., 2014) associated with more severe clinical motor symptoms of FXTAS (McLennan et al., 2022). Based on findings of cerebellar and brainstem pathology in FXTAS, we explored visual gaze fixation and expected increased rates of SWJ would be associated with more severe FXTAS symptoms.

## 2 Method

### 2.1 Participants

Fifty-four participants, including 22 premutation carriers (16 female) and 32 age-matched healthy controls (21 female) completed visually guided saccade (VGS) and antisaccade tests, a clinical evaluation with a movement specialist with expertise in neuromotor disorders of aging (P.K.), and a standardized cognitive assessment (Stanford-Binet 5; See Supplementary Table S1, S2 for medication information). Among these participants, 4 control females did not complete the antisaccade test, 1 female premutation carrier did not complete the antisaccade test, and 1 female premutation carrier did not complete the VGS test. Some participants did not complete all tests due to scheduling issues; tests described here were administered as part of a larger assessment battery designed to assess motor, cognitive, and brain function.

*FMR1* premutation carrier status was confirmed for all participants either by providing the study team with documentation of prior genetic testing, or through molecular testing conducted at the Molecular Diagnostic Laboratory at Rush University. Normal allele status was confirmed for all control participants. DNA was isolated from peripheral blood leukocyte samples, and PCR testing using commercially available Asuragen kits was used to quantify allele-specific CGG repeat length.

### 2.2 Procedure

Participants completed VGS and antisaccade tests in a darkened room, positioned in a chin rest and seated 60 cm from the center of a 102 cm anti-glare LCD monitor (resolution: 1920 × 1,060, refresh rate: 60 Hz). Eye movements were recorded using an infrared, binocular camera-based eye tracking system (500 Hz; EyeLink II, SR Research Ltd., Canada). Before each block of trials, participants performed a nine-point calibration.

#### 2.2.1 Visually guided saccade task (VGS)

A VGS test was administered to examine gaze fixation, saccade latency, and saccade accuracy, as we have done previously (Schmitt et al., 2014). Visual stimuli subtending 0.5° of visual angle were presented in the horizontal plane at eye level. A central fixation was presented for 1.5–2.0 s (varied randomly), after which a peripheral target was presented for 1.5 s at 12° or 24° to the right or left of central fixation. The central fixation did not overlap with presentation of the peripheral target, nor was there a gap between the central fixation and peripheral target without a visual stimulus. Blocks of 30 trials were administered for each target step amplitude (12° and 24°), with direction (left or right) and amplitude pseudorandomized so that there were an equal number of trials at each of the four possible locations. Participants were instructed to look at peripheral targets as quickly as possible.

Saccade onset and offset were marked when velocity exceeded or fell below 30°/sec, respectively. Saccade latencies were defined as the interval between the appearance of the peripheral target and initiation of the primary saccade. To assess accuracy, saccade gain was measured, defined as the ratio of the saccade amplitude to the target step amplitude. SWJ during periods of fixation preceding and following saccades were counted and defined as fixation intrusions in which individuals made two saccades that were of relatively equal size, between 0.20° and 3.0°, in opposite directions of each other, and occurring within 100–400 ms of one another (Nowinski et al., 2005). During the first saccade of each SWJ, gaze shifted away from the fixation target, and during the second saccade, gaze returned to the target.

#### 2.2.2 Antisaccade task

The antisaccade test measured individuals’ ability to inhibit saccades toward a peripheral target. Stimulus parameters were like the VGS test, though participants completed gap and overlap conditions. During gap trials, peripheral target onset occurred 200 ms after removal of the central fixation. During overlap trials, peripheral target onset occurred 200 ms prior to central fixation removal. Participants were instructed to inhibit eye movements toward peripheral targets and instead make a saccade to the mirror location of the target in the opposite hemifield. Antisaccade latency and the proportion of trials in which participants looked toward rather than away from the peripheral target (antisaccade error) were examined. Inhibitory cost was calculated as the difference between VGS latency and antisaccade latency with greater values representing greater inhibitory cost, or lower efficiency in inhibiting unwanted saccades (Wong et al., 2014; McLennan et al., 2022).

### 2.3 Clinical measures

To assess clinical neuromotor deficits that are indicative of FXTAS, a movement specialist (P.K.) administered the International Cooperative Ataxia Rating Scale (ICARS) (Trouillas et al., 1997) which has a maximum score of 100. Nine of the 22 premutation carriers were unable to complete the ICARS due to scheduling issues. Although many participants completed T2 weighted MRI scans, for this study we did not assign FXTAS diagnoses. However, six premutation carriers showed clinical and/or radiological signs consistent with a diagnosis of possible, probable, or definite FXTAS (Supplementary Table S3). An additional eight premutation carriers did not show sufficient signs to warrant a FXTAS diagnosis, whereas eight premutation carriers did not provide sufficient clinical or MRI data to make a diagnostic determination (Hagerman et al., 2001, 2004). For our analyses, we considered all premutation carriers together regardless of FXTAS symptomology but included ICARS total score as a continuous predictor that indicated severity of motor symptoms.

### 2.4 Data analysis

The dependent variables of interest were VGS latency and gain, SWJ, antisaccade latency and error, and inhibitory cost (Table 1). Inhibitory cost was calculated as the difference between VGS latency and antisaccade latency for all combinations of target step amplitude and fixation offset condition. The VGS test did not have separate gap and overlap offset conditions like the antisaccade test, so, for example, “inhibitory cost, gap, 12°” refers to the difference between VGS latency at the 12° step amplitude and antisaccade latency in the gap condition at the 12° step amplitude. When assessing the normality of the dependent variables for model selection, we found that when collapsed across stimulus properties (i.e., target step amplitude, fixation offset condition) all were within limits for little to moderate skew, indicating that general linear models would be acceptable.

**Table 1:** Mean performance on visually guided saccade, antisaccade, and inhibitory cost across *FMR1* premutation carriers and controls.

	Males & Females			Females only		
	Control	*FMR1* Carriers	Hedge’s *g*	Control	*FMR1* Carriers	Hedge’s *g*
***VGS***	*n = 31*	*n = 21*		*n = 21*	*n = 15*	
Latency (12°)	239 (23)	246 (25)	−0.29	245 (23)	245 (24)	0.00
Latency (24°)	250 (24)	260 (25)	−0.41	255 (26)	262 (25)	−0.27
Gain (12°)	0.94 (0.04)	0.95 (0.04)	−0.25	0.95 (0.04)	0.94 (0.03)	0.28
Gain (24°)	0.95 (0.03)	0.94 (0.03)	0.33	0.95 (0.03)	0.95 (0.03)	0.00
***Square Wave Jerks***	*n = 25*	*n = 19*		*n = 17*	*n = 15*	
SWJ Total	19 (13)	19 (15)	0.00	19 (12)	20 (13)	0.08
***Antisaccade***	*n = 28*	*n = 21*		*n = 17*	*n = 15*	
Latency (Gap, 12°)	363 (72)	399 (61)	−0.53	356 (54)	403 (55)	−0.86
Latency (Gap, 24°)	376 (68)	413 (74)	−0.52	370 (62)	415 (69)	−0.69
Latency (Overlap, 12°)	633 (74)	647 (70)	−0.19	633 (75)	660 (73)	−0.36
Latency (Overlap, 24°)	631 (67)	655 (63)	−0.37	631 (67)	671 (63)	−0.61
Error (Gap, 12°)	0.26 (0.19)	0.24 (0.16)	0.11	0.29 (0.17)	0.21 (0.14)	0.51
Error (Gap, 24°)	0.18 (0.15)	0.18 (0.17)	0.00	0.19 (0.14)	0.16 (0.12)	0.23
Error (Overlap, 12°)	0.18 (0.12)	0.21 (0.14)	−0.23	0.21 (0.12)	0.19 (0.15)	0.15
Error (Overlap, 24°)	0.13 (0.12)	0.14 (0.14)	−0.08	0.15 (0.13)	0.11 (0.08)	0.37
***Inhibitory Cost***	*n = 27*	*n = 20*		*n = 17*	*n = 14*	
Gap, 12°	120 (69)	158 (54)	−0.60	112 (51)	165 (49)	−1.06
Gap, 24°	121 (55)	158 (68)	−0.61	115 (55)	159 (62)	−0.76
Overlap, 12°	392 (74)	408 (63)	−0.23	388 (73)	424 (64)	−0.52
Overlap, 24°	378 (54)	411 (51)	−0.63	377 (55)	416 (53)	−0.72

Negative effect sizes indicate the mean was lower in the control group than the premutation group; for example, on antisaccade latency, Gap 12° controls were on average a half standard deviation faster to respond than premutation carriers.

Using PROC GLM in SAS software 9.4 for Windows (SAS Institute Inc, 2013), we examined the predictive effects of stimulus properties, group (control vs. *FMR1* premutation), FXTAS symptom severity/ICARS score, and CGG repeat length. For each dependent variable, we conducted the following models to test the effects of group, ICARS score, and CGG repeat length on oculomotor performance. First, we evaluated a model with only stimulus properties and used an F-test to test whether the total variance accounted for by the model (i.e., R^2^) was significantly greater than zero. For VGS variables, we included a repeated effect of target step amplitude (i.e., 12° vs. 24°), and for antisaccade variables, we included a repeated effect of a four-category dummy variable which represented each combination of target step amplitude and fixation offset condition (i.e., gap vs. overlap). Next, we assessed the effects of group, ICARS score, and CGG repeat length individually while retaining the stimulus property effects, since stimulus properties are known to affect performance and were significant predictors of performance in our models (Supplementary Tables S4, S5). To test the significance of the main effects of predictors, we conducted Wald tests of fixed effects. With this method of quantitative analysis, results can be interpreted as the main effect of the predictor across all levels of stimulus properties on the dependent variables.

We used Hedge’s *g* to calculate effect size for differences between the means because it accommodates uncertainty in small sample sizes. We did not apply multiple comparison (i.e., Bonferroni) corrections, rather we report in full the significance values and effect sizes for all comparisons. We selected this approach given that (1) we were testing pre-planned hypotheses, (2) findings would not contribute to clinical decision-making, and (3) interpretations of findings were not dependent on the number of tests performed (Perneger, 1998; Armstrong, 2014). Finally, we used this same approach in a female only subset of the sample to determine whether the relationships were present within females or within males and females together.

## 3 Results

### 3.1 Descriptives, between-group differences, and stimulus properties

First, we examined group means for all dependent variables (Table 1). Effect sizes based on means and standard deviations for each group’s performance suggested that groups did not differ on VGS latency, VGS gain, SWJ, and antisaccade error. Effect sizes were higher for antisaccade latency and inhibitory cost, suggesting greater differences between groups. When considering the female subset, effect sizes were generally stronger than in the male and female sample for antisaccade and inhibitory cost but were lower for VGS latency and gain.

For the general linear models, we first examined the effect of stimulus properties on performance. For VGS latency, target step amplitude accounted for 6–7% of variation in latency, depending on the makeup of the sample (i.e., males and females vs. females). VGS gain did not vary by stimulus property. Stimulus properties were significantly predictive of antisaccade latency and inhibitory cost, accounting for 77–82% of the variation in oculomotor performance, again depending on the makeup of the sample. Variation accounted for was slightly higher in the female subset for antisaccade latency and inhibitory cost. Stimulus properties accounted for 8–10% of variation in antisaccade error.

**Figure 1:**
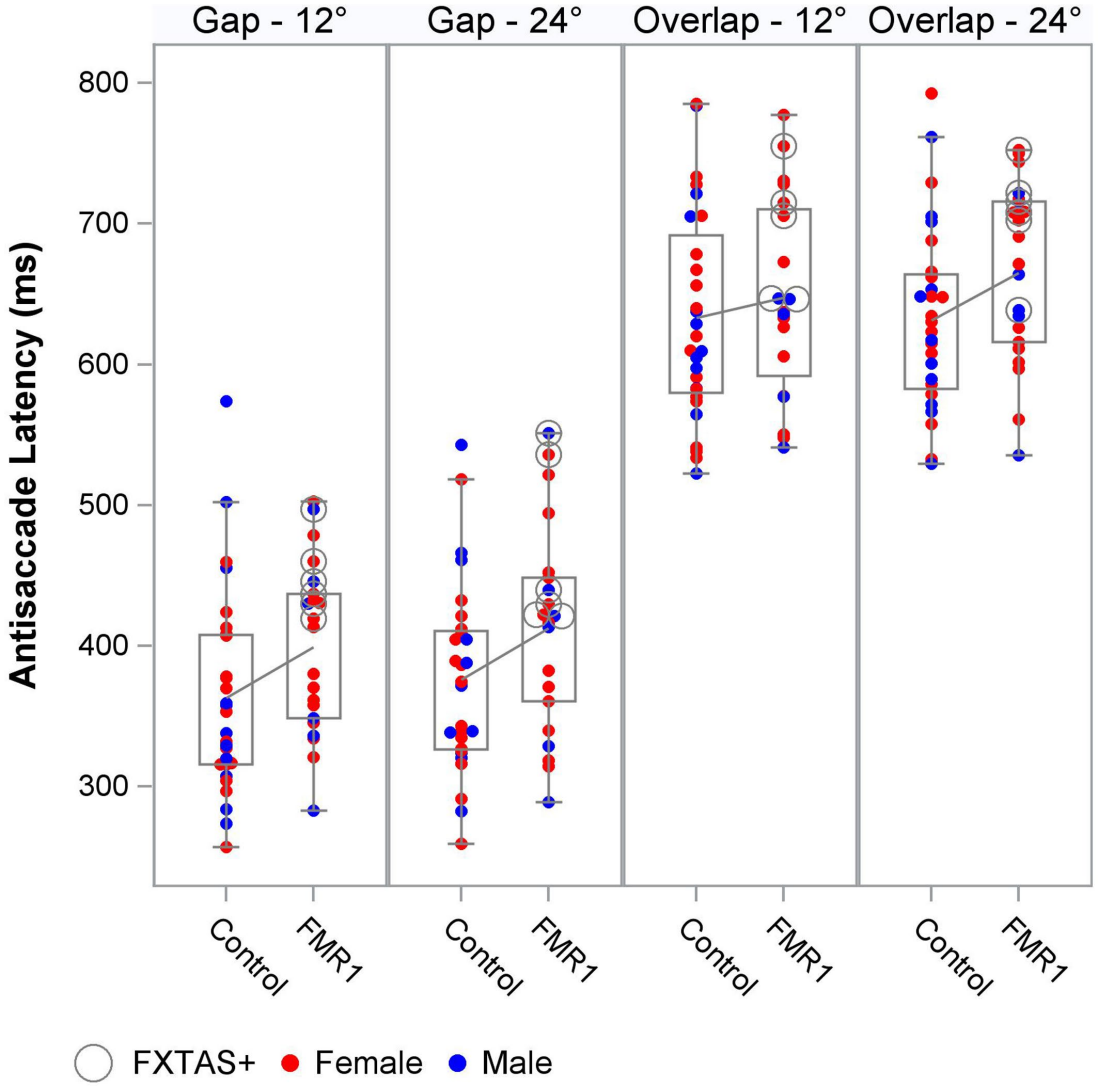
Antisaccade latency distribution by group, target step amplitude, and fixation offset condition.

We next tested the effect of group on oculomotor performance, finding that there were no differences between controls and premutation carriers in VGS latency and gain. Again, effects were similar for both males and females and the female subset. There was a significant main effect of group across all stimulus parameters for antisaccade latency and inhibitory cost for both the male and female sample and the female subset (Figure 1), longer latencies [F_males + females_ (1,190) = 9.4, *p* = 0.003, ΔR^2^ = 2%; F_females_ (1,123) = 11.9, *p* = 0.001, ΔR^2^ = 2%] and longer inhibitory costs [F_males + females_ (1,182) = 11.4, *p* = 0.001, ΔR^2^ = 1%; F_females_ (1,119) = 16.7, *p* = 0.001, ΔR^2^ = 3%] for premutation carriers, with R^2^ increasing slightly to 79–85%. There was no effect of group on antisaccade error.

### 3.2 FXTAS symptom severity and CGG repeat length

**Figure 2:**
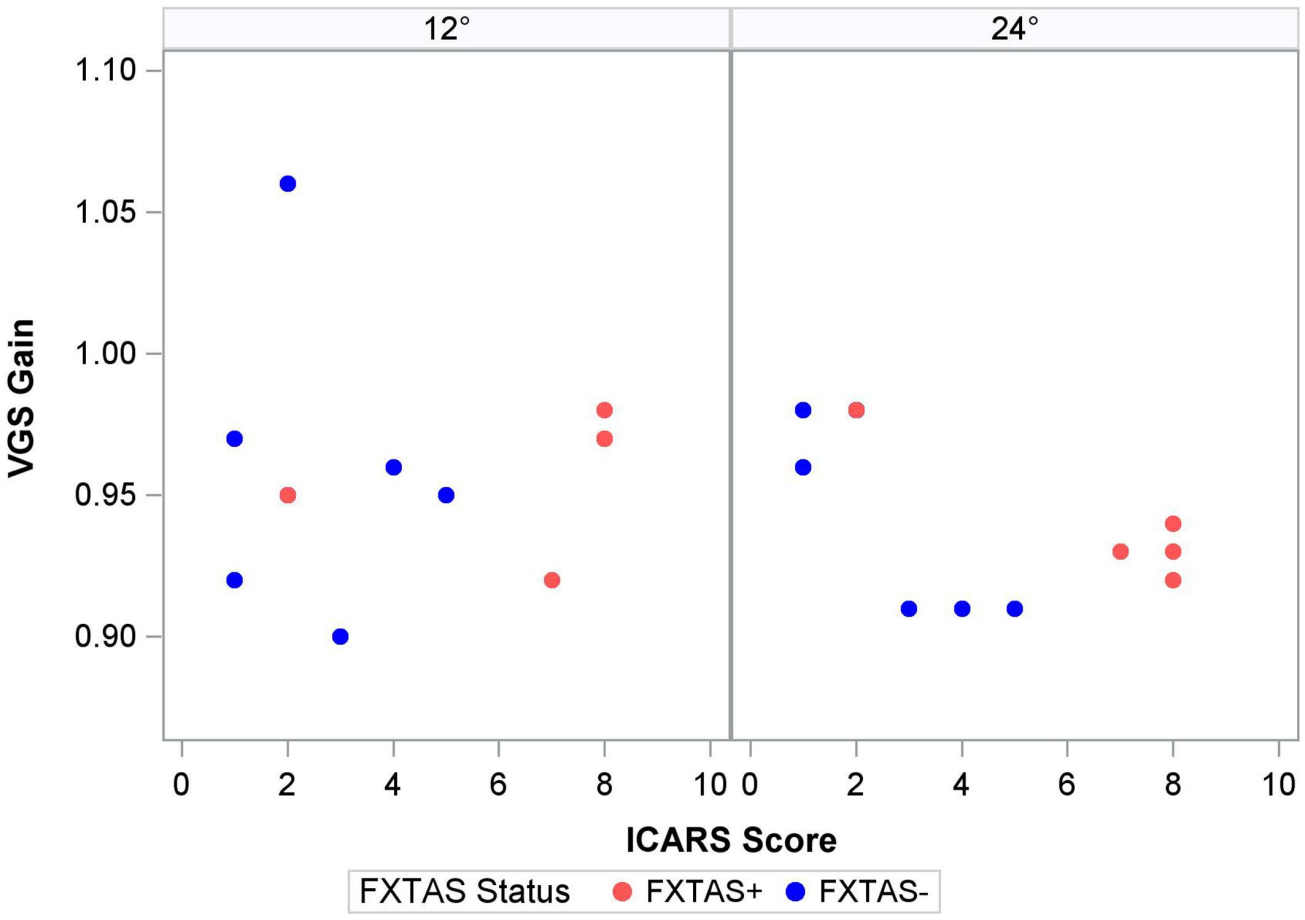
Saccade gain and ICARS score by target step amplitude.

**Figure 3:**
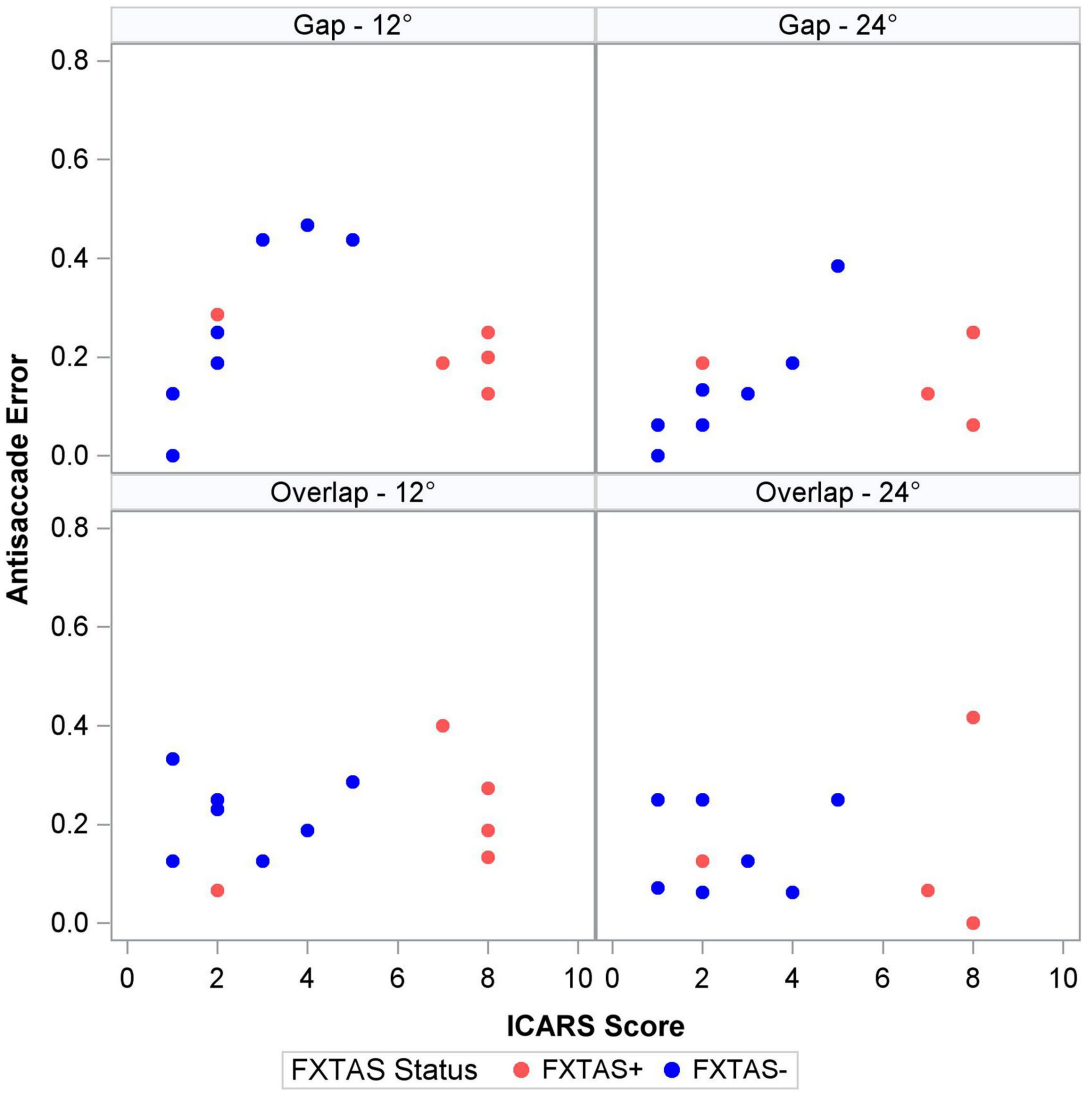
Antisaccade error and ICARS score by target step amplitude and fixation offset condition.

Finally, within the premutation carriers, we tested the effects of FXTAS symptom severity, as measured by the ICARS score, and CGG repeat length. Increasing FXTAS symptom severity was significantly predictive of more severe saccade undershooting (i.e., hypometria on the VGS test; Figure 2) in the male and female sample [*F* (1,25) = 8.6, *p* = 0.007, ΔR^2^ = 27%], but not the female-only subsample [F(1,17) = 2.6, *p* = 0.13, ΔR^2^ = 16%]. Similarly, increasing ICARS score was predictive of increasing antisaccade error in the male and female sample [*F* (1,51) = 22.2, *p* < 0.0001; Figure 3] which accounted for an additional 26% of the variation in antisaccade error. This association was marginally significant in the female-only subsample [*F* (1,31) = 3.4, *p* = 0.08, ΔR^2^ = 7%]. Higher CGG repeat length was associated with increased VGS latency in the male and female sample [*F* (1,31) = 5.6, *p* = 0.02, ΔR^2^ = 18%], but not the female subset [*F* (1,19) = 2.0, *p* = 0.16, ΔR^2^ = 17%]. CGG repeat length was associated with antisaccade latency in the male and female sample such that higher CGG repeat length predicted longer antisaccade latency [*F* (1,66) = 13.4, *p* < 0.001, ΔR^2^ = 4%], but again this was marginally significant in the female-only subset [*F* (1,43) = 3.1, *p* = 0.09, ΔR^2^ = 1%]. When examining the SEs for estimates in the SWJ models, it became clear that these models were underpowered, and although the models were estimable, they were unreliable.

## 4 Discussion

This study represents the first known quantitative assessment of oculomotor behavior in a primarily female sample of aging *FMR1* premutation carriers. While saccadic behavior was similar across premutation carriers and controls, inhibitory control of saccades was disrupted in *FMR1* premutation carriers relative to controls, suggesting top-down control systems may be selectively impacted in males and females with the *FMR1* premutation. Associations between both saccade accuracy and antisaccade accuracy with clinical symptoms of FXTAS indicate that sensorimotor and inhibitory control abilities may track with disease progression. In the context of our sample being relatively mildly affected as indicated by their low ICARS ratings and the ability for all of them to walk without assistance or balance issues, these results suggest that assessment of visually guided saccades and antisaccades may be useful for tracking FXTAS symptom onset and progression in both males and females. Also, given the small number of males in our sample (*n* = 6), and the similarity of findings when examining males and females together and females alone, these results suggest that oculomotor outcomes may be sensitive to disease symptomology in females as well.

Prior studies of saccade behavior in asymptomatic premutation carriers have documented mixed findings suggesting both impaired and spared latencies and preserved accuracy (Shelton et al., 2014; Wong et al., 2014; Moser et al., 2021). Recently, McLennan et al. extended these findings by including FXTAS+ premutation carriers in a primarily male sample and showed that saccade accuracy was more variable in FXTAS patients compared to asymptomatic premutation carriers, though this effect did not survive corrections for multiple comparisons (McLennan et al., 2022). Here, we focus on a predominantly female sample and show that saccade latency and accuracy each appear to be unaffected during initial disease stages. There was a strong association between saccade accuracy (i.e., VGS gain) and severity of motor symptoms in premutation carriers, indicating that saccade behavior may track with disease severity or progression in both sexes. This association was specific to larger amplitude saccades during which more severely clinically affected premutation carriers showed greater hypometria, indicating more severe impairment when the demands on the motor system are increased (Figure 2). This finding may help explain the absence of group differences in saccade accuracy between FXTAS patients and asymptomatic premutation carriers reported in a prior study that examined smaller target step amplitudes (4.5°) (McLennan et al., 2022). Studies of visually guided saccades across a larger range of target step amplitudes may be informative for understanding how saccade accuracy is affected in premutation carriers and to what extent it tracks with aging and/or disease progression.

Antisaccade latency was prolonged in individuals with the *FMR1* premutation, supporting assertions that inhibitory control is affected in male and female premutation carriers. Specifically, our findings are consistent with results from studies of males with FXTAS (McLennan et al., 2022), and extend previous findings in premutation females (Shelton et al., 2014; Moser et al., 2021) by including females with clinically rated symptoms of FXTAS. Prolonged antisaccade latencies could precede reductions in accuracy as suggested by associations between ICARS score and antisaccade latency (though non-significant) and accuracy in premutation carriers (Figure 3), and prior findings that antisaccade accuracy is impaired in more severely affected FXTAS patients (McLennan et al., 2022). Our finding that antisaccade latencies were elevated in premutation carriers relative to controls in the absence of differences in visually guided saccade latencies also indicates that executive function deficits may precede or be more severe than basic motor impairments in male and female premutation carriers who go on to develop FXTAS. Indeed, neuropsychological tests of executive function show that deficits in inhibitory control track with FXTAS development and severity (Famula et al., 2022), and that declines in executive function may be early indicators of FXTAS development and/or risk in males (Cornish et al., 2009; Hashimoto et al., 2011; Grigsby et al., 2014). Deficits in inhibitory control are common in premutation females (Kraan et al., 2013a,b; Klusek et al., 2020; Moser et al., 2021), and it is likely that executive deficits also are early indicators of FXTAS development in females (Kraan et al., 2013a,b; Yang et al., 2013). Longitudinal studies of disease progression in females, including motor and executive deficits, are urgently needed.

### 4.1 Significance for biomarker identification in FXTAS

In this study, we selected oculomotor measures based on their sensitivity to motor and executive function degeneration in diseases of aging as well as their highly quantitative and translational nature. This approach allows us to generate inferences regarding brain mechanisms underlying symptoms of FXTAS. White matter hyperintensities of the MCP are a major radiological feature of FXTAS (Jacquemont et al., 2003), and cerebellar atrophy and white matter disease are common (Greco et al., 2002; Hagerman et al., 2003; Jacquemont et al., 2003; Greco et al., 2006; Tassone et al., 2012; Ariza et al., 2016). Considering that numerous studies document linkages between cerebellar and brainstem pathology and atypical saccade dynamics and precision in humans and non-human primates (Takagi et al., 1998; Leigh and Zee, 2006; Krauzlis et al., 2017), we hypothesized reduced saccade precision in carriers with elevated ICARS scores. Our results linking FXTAS symptom severity and saccade accuracy are consistent with multiple studies of FXTAS showing cerebellar-brainstem circuit degeneration (Greco et al., 2006; McKinney et al., 2020; Famula et al., 2022), suggesting that saccade dysmetria may occur in both male and female carriers as symptoms of FXTAS progress. Given that the MCP sign is rare in females (and was confirmed in only one of our female participants), it is possible that cerebellar mechanisms of sensorimotor function are relatively spared, and that our finding of associations between reduced saccade accuracy and clinically rated motor signs reflects early stages of cortical or striatal atrophy (Adams et al., 2007), perhaps involving white matter degeneration including the splenium of the corpus callosum that often is affected in females with FXTAS (Schneider et al., 2020) and involved in visual to motor processes supporting oculomotor control (Blaauw and Meiners, 2020).

Increased antisaccade latencies in carriers with symptoms of motor degeneration implicate impairment in top-down processes supported by fronto-parietal and fronto-striatal circuits (Shelton et al., 2014; Wong et al., 2014; Moser et al., 2021; McLennan et al., 2022), which is consistent with findings of structural and functional changes within these circuits in FXTAS (Hashimoto et al., 2011). Increased antisaccade latencies in carriers with clinical FXTAS symptoms may reflect degeneration of frontal eye field (FEF) movement cells or their functional communication with dorsolateral prefrontal cortex (DLPFC), as lesions in DLPFC are associated with increased antisaccade errors, and lesions in FEF are associated with longer antisaccade latencies (Pierrot-Deseilligny et al., 1991, 2002, 2003; Ploner et al., 2005). Males with FXTAS show white matter degeneration in frontal and parietal circuits, and females with FXTAS demonstrate intranuclear inclusions in frontal cortex along with frontal lobe atrophy (Tassone et al., 2012). Abnormal frontal P3 ERPs have been reported in females with FXTAS, and reduced activation in prefrontal cortex has been shown in mixed-sex FXTAS samples, both of which are hypothesized to underlie executive dysfunction (Hashimoto et al., 2011; Yang et al., 2013). Additionally, neural hyperexcitability during resting state has been associated with executive dysfunction in asymptomatic female premutation carriers, suggesting that neurophysiological differences that may underlie executive dysfunction are present in asymptomatic premutation carriers (Schmitt et al., 2022). In Parkinson’s disease, abnormal prefrontal alpha and beta oscillations are believed to interfere with proactive response inhibition, leading to longer antisaccade latencies but unaffected antisaccade accuracy (Waldthaler et al., 2022). Together, our findings and previous literature support impairment in prefrontal oculomotor inhibitory circuits in males and females with symptoms of FXTAS.

Increased CGG length was modestly associated with increased antisaccade latencies in premutation carriers during the overlap condition only. In asymptomatic female premutation carriers, curvilinear relationships between CGG length and latency of inhibitory responses have been demonstrated (Klusek et al., 2020), but CGG repeat length has not been found to associate with other cognitive or motor abilities (Storey et al., 2021). In asymptomatic male premutation carriers, longer CGG repeat length has been associated with larger inhibitory cost, but this association was weak (*r* = 0.23, *p* = 0.01) and statistical significance was influenced by which allele was considered in a single mosaic individual (Wong et al., 2014). Larger sample studies assessing non-linear relationships between CGG repeat length, activation ratio for females, and separate molecular mechanisms implicated in FXTAS (e.g., mRNA transcript and FMR protein) are needed to determine mechanistic pathways contributing to FXTAS onset and degeneration.

### 4.2 Limitations

Although the findings here provide new information on sensorimotor and inhibitory control processes in *FMR1* premutation carriers, there are several limitations to note. First, the sample size was small, and groups were uneven in number, limiting statistical power. Analyses of effect sizes identified multiple medium-to-large group differences and associations between oculomotor behaviors and clinical symptoms. Larger samples integrating a broader range of clinical impairments and full diagnostic information among males and females with FXTAS are needed. Second, many of the *FMR1* carriers did not have ICARS assessment scores, and those who did have ICARS scores had low levels of impairment, which limits the generalizability of clinical associations. Longitudinal examination will be necessary to clarify neurodegeneration and development of FXTAS symptoms in currently asymptomatic premutation carriers, as well as to determine changes in penetrance rates as a function of age in female premutation carriers. Finally, although oculomotor behavior may be a robust biomarker, there are cost and time limitations that might impede the scalability or clinical implementation of oculomotor tests as indicators of disease development and severity.

### 4.3 Conclusion

In this study, we examined a predominantly female sample of premutation carriers with and without motor degeneration. Female premutation carriers with FXTAS are a highly understudied population, and little is known about key phenotypes for clinical tracking and assessing degenerative processes. Our findings show that saccadic behavior may not be impacted in females with mild symptoms of FXTAS, but saccade hypometria may track with moderate FXTAS symptoms. Additionally, we demonstrated that females with symptoms of FXTAS have deficits in inhibitory control that also track with disease progression, suggesting executive impairments may represent early indicators of FXTAS in females and serve as sensitive, quantitative, and highly translational biomarkers of disease progression.

## Data availability statement

The raw data supporting the conclusions of this article will be made available by the authors, without undue reservation.

## Ethics statement

The studies involving humans were approved by UT Southwestern Medical Center IRB. The studies were conducted in accordance with the local legislation and institutional requirements. The participants provided their written informed consent to participate in this study.

## Author contributions

HF-G: Conceptualization, Formal analysis, Writing – original draft, Writing – review & editing. SK: Conceptualization, Investigation, Writing – review & editing. KU: Conceptualization, Writing – review & editing. LS: Conceptualization, Investigation, Project administration, Writing – review & editing. SP: Investigation, Project administration, Writing – review & editing. PK: Investigation, Project administration, Writing – review & editing. MM: Conceptualization, Formal Analysis, Funding acquisition, Investigation, Project administration, Writing – review & editing.
