# Supplementary material for: Sensorimotor and inhibitory control in aging FMR1 premutation carriers
Source: Front Hum Neurosci. 2023 Nov 16;17:1271158. doi: 10.3389/fnhum.2023.1271158 (PMC10687573; doi:10.3389/fnhum.2023.1271158)
Supplement: Supplementary file 1 [file Table_1.docx]

**Supplementary Table 1: Participant Demographics**

|  | **Control** | ***FMR1 Carriers*** |
| --- | --- | --- |
|  | *n=32 (21 females)* | *n=22 (16 females)* |
| **Age (SD)** | 52.2 (8.6) | 55.5 (8.3) |
| **CGG Length (range)** | -- | 85 (55-131) |
| **ICARS Score (range)** | -- | 5 (0-12) |
| **Full Scale IQ(SD)** | 107 (14) | 100 (12) |
| **Race** | Asian = 1  Black = 4  White = 27 | Asian = 1  Black = 0  White = 21 |
| **Ethnicity** | Hispanic/Latino = 6  Not Hispanic/Latino = 26 | Hispanic/Latino = 2  Not Hispanic/Latino = 22 |

**Supplementary Table 2: Participant Medications by Sex and Group**

|  | Controls | | *FMR1* | |
| --- | --- | --- | --- | --- |
|  | Female | Male | Female | Male |
|  | n=21 | n=11 | n=16 | n=6 |
| Antihypertensives | 3 | 2 | 6 | 3 |
| SSRIs, SNRIs | 0 | 0 | 5 | 1 |
| Beta blockers | 0 | 0 | 4 | 1 |
| Statins | 6 | 0 | 1 | 3 |
| Thyroid hormones | 3 | 0 | 3 | 1 |

**Supplementary Table 3: Clinical and Radiological Reports for *FMR1* Premutation Carriers**

|  | Sex | CGG Repeats | Age (years) | ICARS Total | MRI Findings | Neurological exam | Classification |
| --- | --- | --- | --- | --- | --- | --- | --- |
| 1 | Female | 102 | 62 | 0 | Not collected | Mild tremor, head titubation | N/A |
| 2 | Female | 99 | 54 | 7 | No MCP sign,  No WM lesions,  No cerebral atrophy | Mild cerebellar features,  Mild tremor | FXTAS+ |
| 3 | Female | 92 | 46 |  | No MCP sign,  No WM lesions,  No cerebral atrophy | No tremor,  No gait ataxia | FXTAS- |
| 4 | Female | 87 | 55 | 2 | No MCP sign,  WM lesions,  Cerebral atrophy type 1 | No tremor,  No gait ataxia | FXTAS- |
| 5 | Female | 107 | 59 | 8 | Not collected | Kinetic tremor,  Ataxia | FXTAS+ |
| 6 | Female | 79 | 49 |  | Not collected | Not collected | N/A |
| 7 | Female | 131 | 39 |  | Not collected | Not collected | N/A |
| 8 | Female | 99 | 51 |  | Not collected | Not collected | N/A |
| 9 | Female | 102 | 61 | 3 | No MCP sign,  Generalized WM lesions,  Cerebral atrophy type 2 | No tremor,  No gait ataxia | FXTAS- |
| 10 | Male | 85 | 71 | 12 | MCP sign,  WM lesions | Tremor,  Gait ataxia | FXTAS+ |
| 11 | Male | 60 | 58 | 4 | No MCP sign,  No WM lesions | No tremor  No gait ataxia | FXTAS- |
| 12 | Male | 63 | 58 | 2 | No MCP sign,  No WM lesions | Tremor,  No gait ataxia | FXTAS- |
| 13 | Female | 62 | 67 | 2 | No MCP sign,  Mild WM lesions,  Dot-like WM hyperintensities | Kinetic tremor,  No gait ataxia  Mild neuropathy | FXTAS+ |
| 14 | Male | 58 | 65 | 8 | Not collected | Kinetic tremor,  Gait ataxia | FXTAS+ |
| 15 | Female |  | 47 |  | Not collected | Not collected | N/A |
| 16 | Female | 110 | 48 | 5 | No MCP sign,  No WM lesions | Tremor,  No gait ataxia | FXTAS- |
| 17 | Female | 68 | 46 | 1 | No MCP sign,  Mild WM lesions,  Dot-like WM hyperintensities | No tremor  No gait ataxia | FXTAS- |
| 18 | Female | 80 | 64 | 1 | No MCP sign,  Mild WM lesions,  Dot-like WM hyperintensities,  Cerebral atrophy type 2 | No tremor,  No gait ataxia | FXTAS- |
| 19 | Male | 64 | 61 |  | Not collected | Not collected | N/A |
| 20 | Female | 78 | 59 |  | No MCP sign,  Mild WM lesions,  Dot-like WM hyperintensities | Not collected | N/A |
| 21 | Female | 81 | 44 |  | No MCP sign,  No WM lesions,  Mild cerebellar atrophy | Not collected | N/A |
| 22 | Male | 93 | 57 | 8 | Suspected MCP sign; Cerebellar and brainstem atrophy | Kinetic tremor,  No gait ataxia | FXTAS+ |

**Supplementary Table 4: General linear models for the effect of stimulus, group, ICARS score, and CGG repeat length on visually guided saccade performance**

|  | VGS Latency | | VGS Gain | | SWJ | |
| --- | --- | --- | --- | --- | --- | --- |
| ***Estimates (SE)*** | M & F | F | M & F | F | M & F | F |
| Intercept | 254 (3.4) ** | 258 (4) ** | .94 (.005) ** | .95 (.01) ** |  |  |
| Condition (24° is reference) |  |  |  |  |  |  |
| 12° | -12 (5) * | -13 (6) * | .001 (.007) | -.003 (.007) |  |  |
| ***Model Fit*** |  |  |  |  |  |  |
| Total R^2^ | .06 * | .07 * | .00 | .002 |  |  |
| F-test (DF) | 6.4 (1,102) | 5.0 (1,70) | .02 (1,102) | .14 (1,70) |  |  |
|  | VGS Latency | | VGS Gain | |  |  |
| ***Estimates (SE)*** | M & F | F | M & F | F | M & F | F |
| Intercept | 259 (4) ** | 260 (5) ** | .94 (.01) ** | .95 (.01) ** | 19.4 (3.2) ** | 19.9 (3.0) ** |
| Group (FMR1 is the reference) |  |  |  |  |  |  |
| Control | -9.0 (5) | -4.0 (6) | .001 (.01) | .01 (.01) | -.01 (4.2) | -.46 (4.2) |
| Condition (24° is reference) |  |  |  |  |  |  |
| 12° | -12 (5) * | -13 (6) * | .001 (.01) | -.003 (.01) |  |  |
| ***Model Fit*** |  |  |  |  |  |  |
| Total R^2^ | .09 ** | .07 | .0002 | .01 | .00 | .00 |
| F-test (DF) | 4.9 (2,101) | 2.5 (2,69) | .01 (2,101) | .36 (2,69) | .00 (1,42) | .01 (1,30) |
|  | VGS Latency | | VGS Gain | |  |  |
| ***Estimates (SE)*** | M & F | F | M & F | F | M & F | F |
| Intercept | 260 (11) ** | 280 (12) ** | .97 (.01) ** | .96 (.01) ** | 11.8 (7.3) | 23.7 (6.1)** |
| ICARS Score | .19 (1.6) | -4.1 (2.5) | -.01 (.001) ** | -.003 (.002) | 1.5 (1.3) | -1.2 (1.4) |
| Condition (24° is reference) |  |  |  |  |  |  |
| 12° | -15 (11) | -23 (13) | .01 (.01) | -.007 (.01) |  |  |
| ***Model Fit*** |  |  |  |  |  |  |
| Total R^2^ | .07 | .27 | .27 * | .16 | .12 | .11 |
| F-test (DF) | .90 (2,25) | 2.8 (2,15) | 4.7 (2,25) | 1.5 (2,15) | 1.4 (1,10) | .72 (1,6) |
|  | VGS Latency | | VGS Gain | |  |  |
| ***Estimates (SE)*** | M & F | F | M & F | F | M & F | F |
| Intercept | 212 (21) ** | 217 (34) ** | .98 (.03) ** | .98 (.04) ** | 5.1 (18.5) | 27 (19) |
| CGG Length | .58 (.24) * | .52 (.37) | -.0005 (.0003) | -.0004 (.0004) | .15 (.22) | -.10 |
| Condition (24° is reference) |  |  |  |  |  |  |
| 12° | -17 (9) | -23 (11) | .008 (.01) | -.005 (.01) |  |  |
| ***Model Fit*** |  |  |  |  |  |  |
| Total R^2^ | .24 * | .24 | .06 | .04 | .04 | .02 |
| F-test (DF) | 4.8 (2,31) | 3.1 (2,19) | 1.0 (2,31) | .40 (2,19) | .48 (1,13) | .23 (1,9) |
| M & F = males and females; F = females only  ** *p* < .01, * *p* < .05 | |  |  |  |  |  |

**Supplementary Table 5: General linear models for the effect stimulus, group, ICARS score, and CGG repeat length on antisaccade performance and inhibitory cost**

|  | Antisaccade Latency | | Antisaccade Error | | Inhibitory Cost | |
| --- | --- | --- | --- | --- | --- | --- |
| ***Estimates (SE)*** | M & F | F | M & F | F | M & F | F |
| Intercept | 646 (10) ** | 650 (12) ** | .13 (.02) ** | .13 (.02) ** | 392 (9) ** | 394 (11) ** |
| Condition (Overlap, 24° is reference) |  |  |  |  |  |  |
| Gap, 12° | -267 (14) ** | -272 (17) ** | .12 (.03) ** | .12 (.03) ** | -256 (13) ** | -259 (16) ** |
| Gap, 24° | -254 (14) ** | -259 (17) ** | .04 (.03) | .04 (.03) | -255 (13) ** | -259 (16) ** |
| Overlap, 12° | -7.0 (14) | -4.0 (18) | .06 (.03) | .07 (.03) | 6.7 (13) | 9.8 (16) |
| ***Model Fit*** |  |  |  |  |  |  |
| Total R^2^ | .77 ** | .80 ** | .08 ** | 0.10 ** | .81 ** | .82 ** |
| F-test (DF) | 219 (3,191) | 163 (3,124) | 5.7 (3,192) | 4.7 (3,124) | 261 (3,183) | 187 (3,120) |
|  | Antisaccade Latency | | Antisaccade Error | | Inhibitory Cost | |
| ***Estimates (SE)*** | M & F | F | M & F | F | M & F | F |
| Intercept | 663 (11) ** | 671 (13) ** | .14 (.02) ** | .11 (.03) ** | 410 (10) ** | 418 (12) ** |
| Group (FMR1 is the reference) |  |  |  |  |  |  |
| Control | -30 (10) ** | -39 (11) ** | -.01 (.02) | .04 (.02) | -31 (9) ** | -43 (11) ** |
| Condition (Overlap, 24° is reference) |  |  |  |  |  |  |
| Gap, 12° | -267 (14) ** | -272 (16) ** | .12 (.03) ** | .12 (.03) ** | -256 (13) ** | -259 (15) ** |
| Gap, 24° | -254 (14) ** | -259 (16) ** | .04 (.03) | .04 (.03) | -255 (13) ** | -260 (15) ** |
| Overlap, 12° | -6 (14) | -4 (16) | .06 (.03) | .07 (.03) * | 7 (13) | 10 (15) |
| ***Model Fit*** |  |  |  |  |  |  |
| Total R^2^ | .79 ** | .82 ** | .08 ** | .12 ** | .82 ** | .85 ** |
| F-test (DF) | 174 (4,190) | 136 (4,123) | 4.3 (4,191) | 4.4 (4,123) | 210 (4,182) | 163 (4,119) |
|  | Antisaccade Latency | | Antisaccade Error | | Inhibitory Cost | |
| ***Estimates (SE)*** | M & F | F | M & F | F | M & F | F |
| Intercept | 665 (22) ** | 689 (23) ** | .05 (.04) | .09 (.05) | 405 (19) ** | 409 (20) ** |
| ICARS Score | 3.8 (2.7) | 3.6 (3.7) | .03 (.01) ** | .01 (.007) | 3.6 (2.4) | 7.7 (3.3) * |
| Condition (Overlap, 24° is reference) |  |  |  |  |  |  |
| Gap, 12° | -268 (26) ** | -278 (28) ** | .10 (.05) | .08 (.06) | -254 (23) ** | -255 (24) * |
| Gap, 24° | -260 (20) ** | -269 (28) ** | .03 (.05) | .01 (.06) | -260 (23) ** | -269 (24) * |
| Overlap, 12° | -23 (27) | -18 (28) | .06 (.05) | .07 (.06) | -7.6 (23) | 5.2 (23) |
| ***Model Fit*** |  |  |  |  |  |  |
| Total R^2^ | .79 ** | .85 ** | .34 ** | .17 | .83 ** | .89 ** |
| F-test (DF) | 47 (4,50) | 45 (4,31) | 6.6 (4,51) | 1.6 (4,31) | 60 (4,50) | 60 (4,31) |
|  | Antisaccade Latency | | Antisaccade Error | | Inhibitory Cost | |
| ***Estimates (SE)*** | M & F | F | M & F | F | M & F | F |
| Intercept | 532 (39) ** | 572 (60) ** | .02 (.09) | -.04 (.10) | 329 (36) ** | 393 (54) ** |
| CGG Length | 1.6 (.43) ** | 1.1 (.63) | .002 (.001) | .002 (.001) | .99 (.40) * | .27 (.57) |
| Condition (Overlap, 24° is reference) |  |  |  |  |  |  |
| Gap, 12° | -268 (21) ** | -271 (27) ** | .10 (.05) | .09 (.05) | -252 (19) ** | -250 (25) ** |
| Gap, 24° | -251 (21) ** | -255 (27) ** | .03 (.05) | .04 (.05) | -252 (19) ** | -256 (25) ** |
| Overlap, 12° | -20 (21) | -13 (27) | .06 (.05) | .08 (.05) | -2.8 (19) | 10 (25) |
| ***Model Fit*** |  |  |  |  |  |  |
| Total R^2^ | .81 ** | .81 ** | .09 | .14 | .84 ** | .85 ** |
| F-test (DF) | 72 (4,66) | 46 (4,43) | 1.7 (4,67) | 1.8 (4,43) | 83 (4,62) | 54 (4,39) |
| M & F = males and females; F = females only  ** *p* < .01, * *p* < .05 | |  |  |  |  |  |

**Supplementary Table 6: Associations between ICARS score and CGG Repeat Length and Oculomotor Performance**

|  | Males & Females | | Females Only | |
| --- | --- | --- | --- | --- |
|  | **ICARS Score** | **CGG Length** | **ICARS Score** | **CGG Length** |
|  | *n=14* | *n=18* | *n=9* | *n=12* |
|  | ρ | *r* | ρ | *r* |
| ***Visually Guided Saccades*** |  |  |  |  |
| Latency (12°) | 0.10 | 0.26 | -0.22 | 0.24 |
| Latency (24°) | -0.17 | **0.52*** | -0.29 | 0.37 |
| Gain (12°) | -0.04 | -0.19 | -0.14 | 0.10 |
| Gain (24°) | **0.68**** | -0.28 | -0.61 | -0.49 |
| ***Square Wave Jerks*** |  |  |  |  |
| SWJ Total | -0.03 | 0.19 | -0.35 | -0.16 |
| ***Antisaccade*** |  |  |  |  |
| Latency (Gap, 12°) | 0.24 | 0.42 | -0.02 | 0.24 |
| Latency (Gap, 24°) | 0.22 | 0.25 | 0.08 | 0.00 |
| Latency (Overlap, 12°) | 0.20 | **0.51*** | 0.37 | 0.42 |
| Latency (Overlap, 24°) | 0.10 | **0.49*** | 0.1 | 0.37 |
| Error (Gap, 12°) | 0.50 | 0.07 | **.067*** | 0.34 |
| Error (Gap, 24°) | **0.68**** | 0.26 | **0.73*** | 0.26 |
| Error (Overlap, 12°) | 0.48 | 0.25 | 0.47 | 0.34 |
| Error (Overlap, 24°) | 0.08 | 0.18 | -0.18 | -0.01 |
| ***Inhibitory Cost*** |  |  |  |  |
| Gap, 12° | 0.24 | 0.35 | 0.26 | 0.04 |
| Gap, 24° | 0.24 | 0.07 | 0.04 | -0.26 |
| Overlap, 12° | 0.19 | 0.46 | **0.69*** | 0.39 |
| Overlap, 24° | 0.26 | 0.38 | 0.42 | 0.19 |

** *p* < .01, * *p* < .05
